# Supplementary material for: Management and Clinical Outcomes of Scleredema Diabeticorum: A Scoping Review
Source: Diseases. 2025 Oct 17;13(10):346. doi: 10.3390/diseases13100346 (PMC12564329; doi:10.3390/diseases13100346)
Supplement: Supplementary file 1 [file diseases-13-00346-s001.zip › diseases-3920195-supplementary.pdf]

Supplementary file

Supplementary Table S1. Full search Strategies used in each database.

Database	Search string
Scopus	(TITLE-ABS-KEY ("scleredema diabeticorum" OR ("scleredema" AND "diabetes")))
MEDLINE (via PubMed)	("scleredema diabeticorum"[MeSH Terms] OR "scleredema diabeticorum"[Title/Abstract] OR ("scleredema"[Title/Abstract] AND "diabetes"[Title/Abstract]))
Cochrane Library	("scleredema diabeticorum" OR ("scleredema" AND "diabetes")) in Title Abstract Keyword

Note. MEDLINE, Medical Literature Analysis and Retrieval System.

Supplementary Table S2. Summary of diabetes type, glycemic status, weight or BMI, insulin regimen, and cutaneous outcomes among patients with scleredema diabeticorum.

Study	Diabetes Type	HbA1c/ Glycemic Control	Weight / BMI	Insulin Regimen / Medications	Comment on Cutaneous Outcome
Llamas-Segura et al., 2025	Type 2	HbA1c 8.6 % → 7.4 % after control	BMI 33 kg/m^2	Metformin + insulin; later added UVA-1 phototherapy	Partial improvement in induration after UVA-1; authors note that metabolic optimization alone was insufficient, but skin texture and flexibility improved after UVA-1 cycles.
Mamadpur & Singh, 2025	Type 2	HbA1c 9.2 % (poor control)	BMI 32 kg/m^2	Oral hypoglycemics + topical emollients + physiotherapy	Partial symptomatic improvement (mobility and stiffness) after physiotherapy; skin induration largely unchanged. Authors emphasize chronic poor glycemic control and long disease duration as contributing factors.
Gracie & Whitaker, 2024	Type 2	HbA1c 10.3 % (persistently high)	BMI 35 kg/m^2	Basal–bolus insulin + metformin	Minimal improvement in skin induration despite partial glycemic control; disease stabilized after introduction of PUVA therapy. Authors note long-standing poor glycemic control and obesity as key contributing factors.
Makary & Razali, 2024	Type 2	HbA1c 10–11%, ↓ to 9.1% after 6 mo	Not stated	Not detailed	Skin thickening unchanged despite improved HbA1c
Ranabahu et al., 2024	Type 2	HbA1c 8.4%, ↓ to 6.8% after 4 mo	BMI 36.3 kg/m^2	Metformin, gliclazide, empagliflozin, sitagliptin	No improvement after glycemic optimization
Waqar et al., 2024	Type 2	HbA1c 9.3%	Not stated	Sitagliptin/metformin	Improved function after phototherapy and counseling for better control
Verma et al., 2022	Type 1	HbA1c 13% improved, then recurrence with HbA1c 16.8%	35 to 33 kg	Insulin (glargine vs mixtard)	Improvement with normoglycemia; recurrence with poor control
Hong et al., 2021	Type 1 + monoclonal gammopathy	Glucose 240 mg/dL (poor control)	Not stated	Insulin (not detailed)	Improvement after IVIG + NB-UVB; glycemic control not central

Kyriakou et al., 2021	Type 1	HbA1c 6.5% (previously poor control)	Not stated	Insulin detemir + aspart	Minimal change despite controlled HbA1c
Linares-González et al., 2021	Type 2	Good metabolic control	Not stated	Not specified	Improved after UVA-1 phototherapy
Ródenas-Herranz et al., 2020	Type 2 diabetes mellitus (> 20 yrs duration)	HbA1c 9.1 % (poor control)	BMI 36 kg/m² (obese)	Basal–bolus insulin + metformin → later UVA-1 phototherapy	Partial improvement after UVA-1; glycemic control alone was insufficient for skin softening. Authors emphasize that phototherapy achieved visible flexibility gain even with persisting hyperglycemia.
Simó-Guerrero 2020	3 Type 1 + 8 Type 2	Mean HbA1c 9.3 % (7.7–14.4) → 2 improved with better control	91 % obese (2 morbid)	91 % on insulin (40 % > 1 IU/kg)	Only partial improvement; 2 improved with weight + metabolic control
Kiyohara 2019	Type 2	HbA1c 7.6 %	Not stated	Glimepiride	Mild case; topical hyaluronidase effective despite moderate HbA1c
Chatterjee 2018	Type 2	HbA1c > 12 % (poorly controlled)	BMI 35.9 kg/m²	Insulin + oral agents	Severe systemic involvement; dysphagia persisted despite treatment
Mickel 2018	Type 2 (newly diagnosed)	HbA1c 9.3 %	BMI 37 kg/m²	Started insulin	Physical therapy improved function but skin unchanged during glycemic stabilization
Sun 2018	Type 2	Chronic and poorly controlled	Not stated	Oral agents	Tranilast improved skin thickness independent of glycemia
Fania 2017	Type 2 (10 yr)	HbA1c 10.1 %, glucose 350 mg/dL	Not stated	Hypoglycemics + insulin	Improved with prednisone + insulin; poor control at presentation
Kennemer 2017	Type 1 (insulin dep.)	HbA1c > 10 % persistently	Not stated	Insulin	IVIG improved skin induration without change in glycemia
Chatterjee 2016	Type 2	HbA1c 7.5 % → improved after weight	BMI 40.6 → ↓ after 16 kg loss	Insulin + oral agents	Skin improved with weight reduction and glycemic optimization

		loss & better control			
Mohamed 2016	New-onset Type 2	Inaugural diabetes, hyperglycemia	Not stated	Insulin started	Improved after insulin + prednisone therapy
Sarı 2016	Type 2	HbA1c 11.8 % (poor)	BMI 35 kg/m^2	Premixed insulin 50 U/d	Partial improvement after insulin + PUVA; notes no clear link between HbA1c and prognosis
Rongioletti 2015	30 DM (26 type 2, 4 type 1)	Mostly poor control (↑glucose, ↑HbA1c)	Obesity frequent	Mixed regimens ± insulin	Strict glycemic control alone improved 1 case; partial in 1 case—otherwise variable
Shazzad 2015	Type 2	HbA1c 8.1 %	Controlle d with insulin	Insulin + MTX + PUVA	Skin and mobility improved; diabetes well controlled
Gandolfi 2014	Type 1	HbA1c 9.3 %	Not stated	Long-term insulin	FREMS therapy improved symptoms independent of HbA1c change
Lin 2014	Type 2	HbA1c 9.6 %	Not stated	Insulin	Persistent skin thickening; no improvement despite control
Shahzad 2014	Type 1	HbA1c 10–11.5 % → 8 % after therapy	BMI 42.6 kg/m^2	Insulin	No skin improvement despite HbA1c improvement
Doğramacı 2012	5 Type 2	HbA1c 8.0 % (mean)	Not stated	Insulin ± OHA	Partial skin improvement with methotrexate, not linked to HbA1c
Kokpol 2012	Type 2	Poorly controlled, long-standing	Not stated	Oral ± insulin	Marked improvement after PUVA + colchicine; independent of glycemic changes
Lee 2011	Type 2	20-yr diabetes	Not stated	Stable insulin	Allopurinol improved skin; no HbA1c change—antioxidant mechanism proposed
Baillot-Rudoni 2006	Type 1 (n=4)	HbA1c ↓ 9.3→7.9 %	Not stated	Implantable pump (intraperitoneal insulin)	Marked improvement in skin and glucose control
Lewerenz 2007	Type 2 (n=3)	Poor control	–	UVA-1 ± antibiotics	Improvement unrelated to HbA1c; early disease responded better
Miyares 2008	Type 2	Poor control	–	Conventional insulin	Fatal case; metabolic control ineffective

Kroft 2008	Type 2 (n=3)	Long-standing, poorly controlled	–	Insulin	Improved with UVA-1, independent of glycemia
Yu 2009	Type 2	New diabetes (hyperglycemia)	–	Insulin	Improvement after radiation, no HbA1c follow-up
Ghosh 2009	Type 1	HbA1c > 8.5 %	29.9 kg/m²	Insulin	Minimal improvement despite control; conservative care
Thumpimukvatana 2010	Type 2 (n=2)	HbA1c 9.5 % / FBS 147 mg dL	–	Insulin or OHA	Good response to UVA-1; HbA1c remained high
Mehta 2010	Type 2	Uncontrolled, improved with insulin	–	Insulin	Mild softening after glycemic improvement
Al-Saeedi & Lee 2010	Type 2 (n=2)	HbA1c 8.5 %	Obese	Insulin ± oral agents + tamoxifen	Marked skin softening; correlated partly with improved DM control
Martín et al. 2011	Type 2	HbA1c 10.2 %	Obese	Insulin	Partial improvement after UVA-1 + weight loss
Meguerditchian et al., 2006	Type 1 (HbA1c 8–9.6%)	Poor control, improved to ~8%	BMI 27	Basal–bolus insulin	No skin change despite partial improvement of HbA1c
Nakajima et al., 2006	2 Type 2	Poorly controlled	–	Oral agents ± PUVA	Marked improvement after PUVA; unrelated to glycemia
Breuckmann et al., 2005	7 Type 2	Mean HbA1c 8.0% (6.7–9.9%)	Mean BMI 37.3	Insulin ± MTX	No improvement despite MTX and physiotherapy; persistent poor control
Gruson & Franks, 2005	Type 1	Long-standing IDDM	–	Insulin	Partial improvement with colchicine, DMSO, and strict glucose control
Tsunemi et al., 2005	Type 2	HbA1c 10.7 %	–	Oral agents	No treatment; chronic lesions persisted; mechanical stress possible cofactor

Note. BMI, body mass index; HbA1c, glycated hemoglobin; IVIG, intravenous immunoglobulin; MTX, methotrexate; NB-UVB, narrowband ultraviolet B; OHA, oral hypoglycemic agents; PUVA, psoralen + ultraviolet A; SD, scleredema diabeticorum; UVA-1, long-wave ultraviolet A.

Supplementary Table S3. Modality-specific overview of dosing parameters, safety, and follow-up outcomes.

Modality	Typical regimen	Typical course (sessions/cycles)	Typical cumulative dose / duration	Adverse events	Durability / relapse
PUVA	Oral/bath PUVA (8-MOP + UVA 30–60 J/cm²)	2–3×/wk; ~20–40 sessions	Often reported as total J/cm² or weeks	Mild erythema/tanning/burning in a subset; no serious events	Responses often sustained for months; occasional relapse after cessation
UVA-1	30–60 J/cm² per session	3–5×/wk; 12–24 sessions	~1,200–1,800 J/cm² when reported	Transient erythema/tanning in some reports; no serious events	Sustained 3–12 months in reports; relapse uncommon while maintained
NB-UVB	Standard NB-UVB dosing	2–3×/wk	Duration-based	Typically, none or mild erythema; no serious events	Short- to mid-term benefit where reported
IVIG	2 g/kg per cycle over 2–5 days	~q4 weeks; 3–≥10 cycles	~6–≥20 g/kg	Headache in one patient; no serious events.	Maintained on therapy; relapse 2–3 months after stopping in one case; re-response on restart
Electron-beam / RT	e^- 20–24 Gy (2 Gy × 10–12 fx) or VMAT 20 Gy/10 fx	Usually single course; re-irradiation in select cases	20–24 Gy per course	Grade 0–1 skin changes; no significant toxicity	Relief for months; lower-dose courses less durable; re-irradiation feasible
Tamoxifen	20 mg bid → 20 mg qd	Up to ~18 months	Duration-based	Vaginal bleeding (led to discontinuation)	Relapse after cessation noted
Tranilast	0.3 g/day	~3 months	Duration-based	NR	Continued improvement during observed period
Methotrexate	5–25 mg/week (once weekly)	≥3 months	Duration-based	Generally, well tolerated in included reports	Short- to mid-term data; longer-term outcomes limited

Note. AE, adverse event; bid/qd, twice / once daily; CR/PR/NR, complete / partial / no response; fx, fractions; Gy, Gray; IVIG, intravenous immunoglobulin; J/cm², joules per square centimeter; MOP, methoxypsoralen; NB-UVB, narrowband ultraviolet B; PUVA, psoralen plus UVA; UVA-1, ultraviolet A (340–400 nm); VMAT, volumetric-modulated arc therapy; NR, none reported. Full citations are provided in the main Reference list; no separate bibliography is included for Supplementary Tables.
